# Pollen transfer in fragmented plant populations: insight from the pollen loads of pollinators and stigmas in a mass‐flowering species

**DOI:** 10.1002/ece3.2280

**Published:** 2016-07-21

**Authors:** Chloé E. L. Delmas, Thomas L. C. Fort, Nathalie Escaravage, André Pornon

**Affiliations:** ^1^ UMR SAVEINRA, BSA, Univ. Bordeaux33882Villenave d'OrnonFrance; ^2^ Lab Evolution & Diversité Biologique EDBUniversité Toulouse III Paul SabatierF‐31062ToulouseFrance; ^3^ CNRSEDBUMR 5174F‐31062ToulouseFrance

**Keywords:** Mate limitation, plant density, pollen transport, pollinator limitation, *Rhododendron ferrugineum*, self‐pollination

## Abstract

Pollinator and/or mate scarcity affects pollen transfer, with important ecological and evolutionary consequences for plant reproduction. However, the way in which the pollen loads transported by pollinators and deposited on stigmas are affected by pollination context has been little studied. We investigated the impacts of plant mate and visiting insect availabilities on pollen transport and receipt in a mass‐flowering and facultative autogamous shrub (*Rhododendron ferrugineum*). First, we recorded insect visits to *R. ferrugineum* in plant patches of diverse densities and sizes. Second, we analyzed the pollen loads transported by *R. ferrugineum* pollinators and deposited on stigmas of emasculated and intact flowers, in the same patches. Overall, pollinators (bumblebees) transported much larger pollen loads than the ones found on stigmas, and the pollen deposited on stigmas included a high proportion of conspecific pollen. However, comparing pollen loads of emasculated and intact flowers indicated that pollinators contributed only half the conspecific pollen present on the stigma. At low plant density, we found the highest visitation rate and the lowest proportion of conspecific pollen transported and deposited by pollinators. By contrast, at higher plant density and lower visitation rate, pollinators deposited larger proportion of conspecific pollen, although still far from sufficient to ensure that all the ovules were fertilized. Finally, self‐pollen completely buffered the detrimental effects on pollination of patch fragmentation and pollinator failure. Our results indicate that pollen loads from pollinators and emasculated flowers should be quantified for an accurate understanding of the relative impacts of pollinator and mate limitation on pollen transfer in facultative autogamous species.

## Introduction

The ecological and evolutionary impacts of pollination failure on plant populations depend on the extent to which pollen transport and receipt are affected by low pollinator availability and/or low conspecific plant availability (Holsinger 1996; Goodwillie et al. 2005; Eckert et al. 2010). Understanding the processes by which pollinator and mate limitations affect plant reproduction has thus become a major scientific challenge, especially as plant and pollinator populations are endangered by global changes (Aguilar et al. 2006; Bjerknes et al. 2007; Hegland et al. 2009).

Plant mate and pollinator limitations may occur independently (e.g., Campbell and Husband 2007; Wagenius and Lyon 2010) or together, because habitat disturbance can decrease both flowering plant density and pollinator populations. Such situations may have various consequences for the pollen transported and deposited by pollinators (hereafter referred to as “pollen transfer”) and subsequent pollen limitation (Eckert et al. 2010; Thomann et al. 2013). For example, Eckert et al. (2010) hypothesized that pollinator limitation, when occurring without mate limitation, likely affects pollen load size (including conspecific and heterospecific pollen), resulting in high mean pollen limitation driven by insect visit scarcity, whereas mate limitation, when occurring without pollinator limitation, likely affects the proportion of conspecific pollen in the pollen load, also resulting in high mean pollen limitation but driven by low mate density.

In generalist pollination system, reduced plant mate availability may lead pollinators to visit co‐flowering species more often and carry a diverse assemblage of pollen from different species. This trend may result in the deposition of only small amounts of conspecific pollen on stigmas, the deposition of heterospecific pollen on the stigma and the loss of conspecific pollen to foreign stigmas, all of which decrease plant reproductive output (Wilcock and Neiland 2002; Morales and Traveset 2008). In this context, specific pollen placement on the body of the pollinator may facilitate conspecific pollen deposition and counteract the negative effect of diverse pollen loads transported by insects (Armbruster et al. 1994; Pauw 2006). At higher plant mate availabilities, there is a tendency for visitors to be more specialized or constant (Kunin 1993, 1997), resulting in the delivery of a higher proportion of conspecific pollen (Thomson 1980; Chittka et al. 1999). However, in large and dense populations or mass‐flowering species, the availability of pollinators can become limited and competition between plants for insect visits may decrease the quantity of pollen transferred and increase the variability of pollen load size (Eckert et al. 2010). Pollinator limitation alone may also cause a similar pattern (Eckert et al. 2010).

The structure and composition of plant and pollinator communities and pollinator behavior directly affect the external pollen load of the pollinator and the pollen load deposited on the stigma by pollinators. Studies of insect pollen loads can provide essential information about plant–pollinator interactions in the community (Alarcón 2010; Popic et al. 2013), and studies of stigma pollen loads should provide information about the pollination services provided (Geber and Moeller 2006; Popic et al. 2013). However, the correlation between stigma and pollinator pollen loads may be only very weakly correlated, particularly in hermaphrodite species. In such species, stigma pollen loads also depends on both self‐pollination capacity (Duncan et al. 2004a) and mechanisms preventing the transfer of heterospecific pollen from the body of the pollinator to the stigma (Armbruster et al. 1994). In hermaphrodite self‐compatible species, determination of the relative contributions of pollinators and autonomous self‐pollination to pollen receipt is crucial and challenging, as self‐fertilization has significant genetic, demographic, and evolutionary consequences for plant populations (Wright et al. 2013). The relative effects of plant and pollinator availabilities on pollen transport and receipt have rarely been evaluated in natural populations, particularly for self‐compatible species. Moreover, the relationship between stigma pollen receipt and the pollen carried by pollinators has rarely been investigated and remains largely unknown (but see Bartomeus et al. 2008; Briscoe Runquist 2013).

In this study, we investigated the effects of plant density, patch size, and insect visits on the size and quality (conspecific vs. heterospecific pollen) of the pollen loads transported by pollinators (bumblebees) and deposited on emasculated and intact flowers in the wild. We used this framework to investigate the correlation between insect and stigma pollen loads in terms of pollen quantity and quality. Studies of emasculated flowers make it possible to study the impact of pollinator visits on the composition and size of stigma pollen loads, whereas studies of intact flowers can be used to assess the combined effects of pollinator visits and autonomous self‐pollination. We used natural patches of the self‐compatible mass‐flowering shrub *Rhododendron ferrugineum* L. (Ericaceae) of contrasting density and size in the French Pyrenees. Mass‐flowering strategies, resulting in a high concentration of resources and great attractiveness to pollinators (Augspurger 1980, 1981), lead to specific responses of plants and pollinators to changes in the spatial distribution of plants and could lead to particular patterns of pollen transfer (Eckert 2000; Dominguez et al. 2005). In particular, insect visitation rate per flower and *R. ferrugineum* patch size have been shown to be negatively correlated (Delmas et al. 2014b): The highest visitation rate occurred in small sparse patches and the lowest visitation rate in large dense patches of *R. ferrugineum*. We have previously shown that mate limitation in small *R. ferrugineum* patches results in low levels of pollen limitation and high selfing rates, whereas pollinator limitation in large patches results in higher levels of pollen limitation and outcrossing rates (Delmas et al. 2015). However, the ecological factors such as pollen transport underlying seed production and mating system patterns in *R. ferrugineum* patches of varying size and density have yet to be identified. Accordingly, we recorded insect visits and studied pollinator pollen loads (bumblebees; Escaravage and Wagner 2004) and stigma pollen loads from emasculated (pollinator‐mediated pollination) and intact flowers in fragmented *R. ferrugineum* heathland patches. We hypothesized that mate limitation would reduce the quality of pollen loads transported and deposited by pollinators (reduced proportion of conspecific pollen) while pollinator limitation would reduce the quantity of pollen loads transported and deposited by pollinators. Self‐pollination, highlighted in pollen loads from intact flowers, could be able to counteract the effects of pollination failure in *R. ferrugineum* heathland patches.

## Materials and Methods

### Study species and study area

*Rhododendron ferrugineum* is an evergreen shrub with a massive floral display consisting of numerous inflorescences, each containing a mean of 10.8 (6–17) bright red nectariferous tubular flowers (Escaravage et al. 1997; Pornon et al. 1997). The flowering period of the population lasts 20–30 days. Flowers last about 10 days and are initiated the year before they mature (Escaravage et al. 1997). They are protandrous with poricidal anthers and present a stamen dimorphism with an inner whorl of five long stamens and an outer whorl of five short stamens (Escaravage et al. 2001). At the study site, this species displays a mixed mating system (the mean patch outcrossing rate is 51% based on 12 microsatellites) and seed production from self‐fertilized flowers reached 58% that of cross‐pollen‐supplemented flowers on average (pollination experiment, Delmas et al. 2015). Self‐fertilization may occur within flower (spontaneous selfing without pollinators or facilitated selfing by pollinators) or between flowers of the same individual (geitonogamy) (Delmas et al. 2014a, 2015). Outcrossing rates and selfing capacity vary across the species range (Escaravage et al. 1997; Charrier et al. 2014; Delmas et al. 2015). At the study site, a diverse community of hymenopterans, lepidopterans and dipterans visits *R. ferrugineum* flowers (Delmas et al. 2014b). Bumblebees and honeybees (rarely observed here) have been described as the efficient pollinators of *R. ferrugineum* (Escaravage and Wagner 2004).

The study was conducted on a 3‐km² area in the French Central Pyrenees (southern France), near the village of Camurac (42°46′31″N; 01°55′45″E). The altitude of this site ranges from 1550 m to 1750 m a.s.l., and the snow cover usually lasts from late October until May. The *R. ferrugineum* heathland patches studied formed visually distinct aggregations of shrubs embedded in the associated flowering plant community. All *R. ferrugineum* heathland patches present in the 3 km² area in 2010 (*N = *27) were surveyed.

### Plant patch structure

We assessed patch conspecific density, by estimating the median distance between each target shrub and its three nearest conspecific neighbors (i.e., isolation of individual plants in each patch). Patch density was therefore the opposite of mean individual isolation (greater isolation of individuals corresponds to a lower patch density). We found a mean individual isolation per patch ± SEM of 7.8 m ± 1.9, assessed on 105 randomly selected individuals in the 27 heathland patches (2–6 shrubs per patch). For analysis purposes (see pollinator pollen load analyses), patches were further classified in two categories according to the median of the mean individual isolation per patch (5 m). Patches with a mean individual isolation value greater than 5 m were classified as “low‐density patches” (*N = *14), and patches with a mean individual isolation value below 5 m were classified as “high‐density patches” (*N = *13). These two categories have contrasted mean individual isolation: 14 m on average for the low‐density patches and 1 m on average for the high‐density patches. This classification was used to sample bumblebees for pollinator pollen load analyses. The total area of each patch (m²) was estimated from the geographic coordinates of patch perimeters recorded at 5‐m intervals and analyzed in Ozi Explorer (GPS mapping software, version 3.95.4m, Des Newman, Brisbane, Australia). Total patch area ranged from 0.01 to 15.77 ha (1.73 ± 0.38 ha).

### Patch flower visit abundance and visitation rate

We estimated *R. ferrugineum* visit abundance in each patch, by conducting standardized surveys of flower‐visits by insects during the peak of the *R. ferrugineum* flowering period in 2010 (June 1–July 10), according to the method described by Delmas et al. (2014a). Briefly, we surveyed each patch twice over the sampling period, with at least a week between samplings, during relatively sunny and clear conditions favoring insect activity. During each sampling period, one person walked slowly for 15 min along a 50 × 2 m transect (total sampling time of 810 min) randomly placed in the core of the patch and recorded all the visits observed within *R. ferrugineum* flowers. We obtained a number of visits to *R. ferrugineum* for each patch. To estimate visitation rate and infer potential pollinator services, the number of visits observed needs to be scaled by the abundance of *R. ferrugineum* flowers in each patch. We divided the abundance of visits to *R. ferrugineum* by the proportion of coverage by this plant in each patch, to assess visit density as a proxy of visitation rate per flower. We estimated the percentage of *R. ferrugineum* cover per patch from (1) the total area of each patch and (2) the area covered by *R. ferrugineum* within each patch (m²), estimated from the area occupied by the focal shrub in a 400‐m² plot selected at random within the core of the patch. Within patches, the percentage of *R. ferrugineum* cover ranged from 0.18 to 98%.

### Pollinator pollen loads

For the assessment of external pollinator pollen loads, we collected a random sample of the *Bombus* sp. (Hymenoptera) visiting the flowers of *R. ferrugineum,* in patches of low and high densities. *Bombus* sp. is the most abundant genera visiting these flowers, and they are known to be the efficient pollinator of this plant (Escaravage and Wagner 2004). Bumblebees were collected over a period of 1 h per patch, once the transect walks had been completed (described above), in a subset of 18 patches representative of either low‐density patches (*n* = 8) or high‐density patches (*n* = 10 patches). Insects were caught in individual clean vials and frozen until processing in the laboratory. Before pollen load analyses, we identified bumblebees to species level according to a reference collection of insects from the study site identified in 2009 by Pr Pierre Rasmont (University of Mons, Belgium). We randomly collected 68 *Bombus* sp. while foraging within *R. ferrugineum* flowers (*n* = 32 in low‐density patches and *n* = 36 in high‐density patches) belonging to 10 species (see Table S1). Two species were the most abundant, *Bombus wurfleini pyrenaicus* (*N *=* *26) and *Bombus soroeensis lectitatus* (*N *=* *19).

We assessed external pollen loads, by systematically dabbing each insect with a small cube of gelatin–fuchsin (see Dafni 1992) to sample and stain the pollen. The gel was placed on a slide, heated to melting point, and covered with a cover slip after the pollen grains had been separated and spread evenly with a thin pin. We thoroughly cleaned the forceps used between insects. We avoided sampling the pollen storage areas of bumblebees, such as pollen baskets, as these areas contain pollen unlikely to be available for pollination and we assumed that the quality of the pollen on the body of the insect would be similar to that in pollen baskets.

We counted the conspecific *R. ferrugineum* pollen (tetrad pollen) and heterospecific pollen from other plant species flowering at the same time on each slide under a binocular microscope. *Rhododendron ferrugineum* pollen grains were the only tetrad pollen observed (no other Ericaceae with tetrad pollen was flowering during the sampling period). If the slide contained more than 1,000 pollen grains, it was divided into 100 squares of 1 mm² each. We counted and identified pollen grains in seven randomly selected squares and calculated the total number of pollen grains as the mean number of grains per square multiplied by the total number of squares in which pollen grains were found. For each insect, we calculated the total pollen load (absolute number of pollen grains of any species) and pollen load quality (proportion of the total pollen load corresponding to conspecific pollen, regardless of its viability and whether the pollen came from the same or a different conspecific individual). The number of conspecific pollen grains was obtained by multiplying the number of conspecific tetrads counted by four. We did not identify the pollen species other than *R. ferrugineum* present, because this study did not aim to assess pollen diversity.

### Stigma pollen loads

In June 2010, we emasculated bud flowers from two inflorescences on each of the 105 target shrubs by excising the anthers (prior to anthesis), to prevent self‐pollen deposition, thereby making it possible to assess pollen delivery by pollinators. At the end of the flowering period of each individual, we randomly collected one style from each of the two emasculated inflorescences and one style from each of two intact control inflorescences. The styles were stored in 70% ethanol, and the stigmas were then mounted in fuchsin stain (see Kearns and Inouye 1993) on a glass slide. We counted all the pollen grains on each stigma. We differentiated between *R. ferrugineum* pollen tetrads (conspecific pollen) and pollen grains from other co‐flowering species (heterospecific pollen). The number of conspecific pollen grains was obtained by multiplying the number of conspecific tetrads by four.

### Statistical analysis

#### Patch visitation rate

We assessed the effect of patch structure (patch density, patch area, and their interaction; fixed effects) on visitor abundance and visitation rates (count variables), with generalized linear mixed models (GLIMMIX procedure in SAS Software, SAS University Edition, SAS Institute, Cary, NC, USA), using patch identity as a random effect. We used a Poisson link function with an overdispersion component to the variance function. Patch visit density and patch area were log‐transformed to reduce the impact of very large patches (~16 ha) and to facilitate the simultaneous analysis of both these predictive variables within models. We considered patch visit density (visit abundance standardized by *R. ferrugineum* coverage) to be a more relevant proxy for pollination services than visit abundance.

#### Pollinator pollen loads

To test the effect of patch density on pollinator pollen loads from *N = *8 low‐density patches and *N = *10 high‐density patches, we used generalized linear mixed models (PROC GLIMMIX) with appropriate link functions. Separate models were generated for the amount of pollen (total number of pollen grains) and pollen quality (proportion of conspecific pollen) averaged per patch. We used a model with Poisson errors and an overdispersion component to the variance function (Thall and Vail 1990) to analyze pollen quantity (i.e., count data). To model pollen quality, we used a binomial distribution with a logit‐link function (quasi‐likelihood estimation; McCullagh and Nelder 1989). Sample sizes for each of the 10 bumblebee species in low‐ and high‐density patches did not allow an analysis at the species scale. The aim was to compare the effect of patch density on pollen loads transported by bumblebees (overall all species) to the effect of patch density on pollen loads deposited on emasculated and intact flowers.

#### Stigma pollen loads

The effects of visitation rate, patch density, patch area, and their interaction (fixed effects) on mean stigma pollen load per patch were assessed with GLMMs (PROC GLIMMIX), to allow the use of appropriate link functions, as described above for visitor pollen loads. We realized separate analysis for emasculated and intact flowers. For each variable (pollen quantity and pollen quality), we used the Akaike's information criterion (AIC) to compare seven models. The models included visit density, patch density, and patch area, together with their interactions, in pairs or one‐by‐one. We selected the model with the lowest AIC, which provided the most reliable fit to the data (Sakamoto et al. 1986). In addition, we compared stigma pollen loads from the “low‐density patches” and the “high‐density patches” in GLMMs, using the appropriate models and link functions according to the type of distribution, as described above.

#### Relationship between pollinator and stigma pollen loads

We investigated the relationship between stigma pollen load and pollinator external pollen load paired by patch (*n* = 18 patches). We used a GLIMMIX procedure using the appropriate link functions for the count and proportion data, as described above. We weighted the analysis by the number of insects collected in each patch.

## Results

### Patch flower visit abundance and visitation rate

We recorded 428 visits of insects from the Hymenoptera, Diptera, and Lepidoptera to *R. ferrugineum* during transect walks. Hymenopterans, the efficient pollinator of the focal species, delivered the most frequent visits to *R. ferrugineum* (64% of visits, mostly *Bombus* sp.). We identified 10 *Bombus* species (see Table S1), of which *B. soroeensis lectitatus* and *B. wurfleini pyrenaicus* were the most frequently detected (36 and 39% of visits by hymenopterans, respectively).

Overall visit abundance increased significantly with patch density (Table 1), whereas overall patch visitation rate decreased significantly with patch density, depending on patch area (marginally significant density × area interaction, Table 1). When visit abundance and visitation rate were analyzed by insect order, we found only one significant relationship: Hymenoptera patch visitation rate decreased slightly with patch density (*P *=* *0.04; Table 1).

**Table 1:** The effect of patch density, patch area, and their interaction on *Rhododendron ferrugineum* visit abundance and patch visitation rate

	Patch visit abundance		Patch visitation rate	
	*F*_1,25_	*P* value	*F*_1,25_	*P* value
Overall visits				
Patch density	5.51	0.027 (+)	9.42	0.005 (−)
Patch area	0.38	0.542	1.13	0.298
Density × area	2.28	0.143	4.69	0.040 (−)
Hymenoptera				
Patch density	3.05	0.09	4.49	0.04 (−)
Patch area	0.25	0.62	0.58	0.45
Density × area	1.68	0.21	2.69	0.11
Diptera				
Patch density	0.14	0.71	0.53	0.47
Patch area	0.00	0.98	0.04	0.84
Density × area	0.03	0.87	0.03	0.86
Lepidoptera				
Patch density	0.41	0.53	0.86	0.36
Patch area	0.05	0.83	0.00	0.99
Density × area	0.43	0.52	0.86	0.36

Patch density, area, and their interaction were fixed effects (log‐transformed), and patch identity was introduced as a random effect (*N = *26 patches). A Poisson link function with an overdispersion component to the variance function was used. Trends (positive or negative relationship) are shown in parentheses for significant relationships (*P*‐values (<0.05).

### Pollinator pollen load and the impact of patch structure

The average amount of total pollen and proportion of conspecific pollen carried by each insect is indicted in Table 2 for all bumblebee species and the two most abundant species. The two most abundant bumblebees had contrasted pollen loads: *B. wurfleini pyrenaicus* individuals, observed more frequently in small sparse patches, carried larger pollen loads with a lower proportion of conspecific pollen than *B. soroeensis lectitatus,* observed more frequently in large dense patches (Table 2).

**Table 2:** Analyses of the pollen loads of *Rhododendron ferrugineum* pollinators and stigmas

	Total pollen load per insect or stigma		Conspecific pollen (% per insect or stigma)	
	Mean (SEM)	Range	Mean (SEM)	Range
Pollinators				
Total *Bombus* *N = *68 ind	4892 (661.4)	122–26916	55.1 (4.7)	0.2–100
*Bombus wurfleini pyrenaicus* *N = *26 ind	4142.5 (1003.6)	122–20794	25.3 (6)	0.2–100
*Bombus soroeensis lectitatus* *N = *19 ind	3277.2 (554.3)	651–9102	92.8 (1.7)	70–100
Stigmas				
Emasculated flowers *N = *197 stig	258.2 (25.1)	0–2708	83.3 (2.2)	0–100
Intact flowers *N = *195 stig	546.3 (44.8)	0–4738	94.8 (1.2)	0–100

Overall means (±SEM) of total pollen load and the proportion of conspecific pollen are presented, based on individual (per insect or stigma) estimation. Insects were collected while foraging within *R. ferrugineum* flowers. Pollen loads for overall *Bombus* sp. (10 species collected in 18 patches), for the two most abundant *Bombus* species in the study site, for emasculated and intact *R. ferrugineum* flowers (collected in 27 patches) are detailed.

Ind, individual; *N*, number of individual insects or stigmas; SEM, standard error of the mean; stig, stigma.

**Figure 1:**
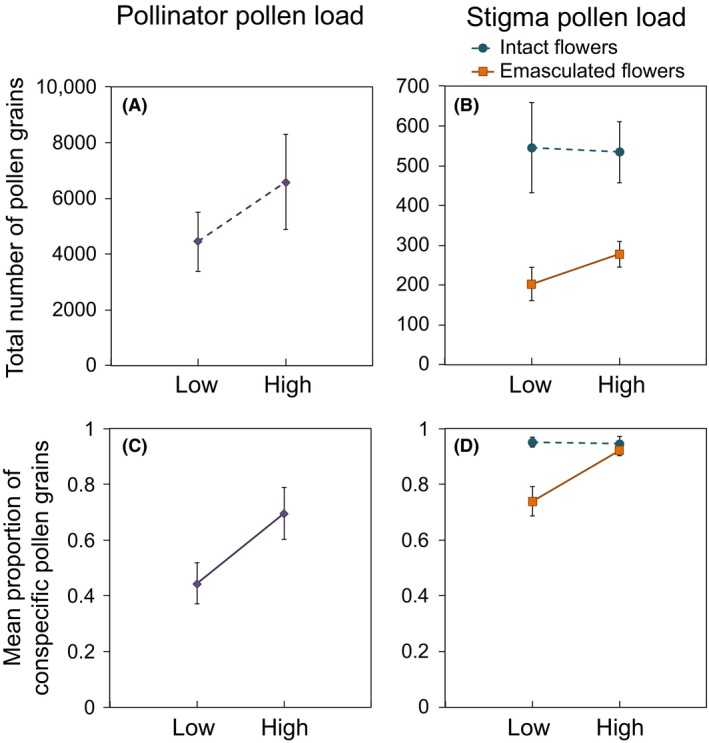
The effect of *Rhododendron ferrugineum* patch density on pollinator and stigma pollen loads: the total amount of pollen transported by pollinators (A; all bumblebees pooled) or deposited per stigma (B) and the mean proportion of conspecific pollen transported per insect (C; all bumblebees pooled) or deposited per stigma (D). In panels B and D, circles and squares represent intact and emasculated flowers, respectively. Low‐density patches are characterized by a mean individual isolation of 14 m and the high‐density patches by a mean individual isolation of 1 m. Solid lines represent significant patch density effects (Table 3).

Patch density (low vs. high) had no significant effect on the mean total number of pollen grains carried by each pollinator (all bumblebees pooled, Fig. 1A, Table 3). The mean proportion of conspecific pollen carried by each pollinator was significantly lower in low‐density patches (all bumblebees pooled, Fig. 1C, Table 3).

**Table 3:** The effect of patch density (low vs. high) on pollen loads of *Rhododendron ferrugineum* pollinators and stigmas

	Total pollen load (per insect or stigma)			Conspecific pollen proportion (per insect or stigma)		
	LDP Mean (CV) (%)	HDP Mean (CV) (%)	Model	LDP Mean (CV) (%)	HDP Mean (CV) (%)	Model
Pollinator						
Overall bumblebees	4446 (68)	6587 (82)	*F*_1,16_ = 0.46 *P *=* *0.51	44.5 (47)	69.5 (42)	*F*_1,16_ = 4.29 *P *=* *0.055
Stigmas						
Emasculated flowers	203 (74)	278 (44)	*F*_1,25_ = 8.54 *P *=* *0.007	73.9 (26)	92 (8)	*F*_1,25_ = 11.15 *P *=* *0.003
Intact flowers	546 (75)	534 (54)	*F*_1,25_ = 0 *P *=* *0.95	95 (6)	95 (10)	*F*_1,25_ = 0.02 *P *=* *0.88

Patch means and coefficients of variation (in parentheses) are presented for low‐density patches (LDP) and high‐density patches (HDP). See Methods section for information about the statistical model for pollen loads. Sample sizes are as follows: *N *=* *32 insects from 8 patches (LDP) and *N *=* *36 insects from 10 patches (HDP) for pollinator pollen loads and *N *=* *13 patches (LDP) and *N *=* *14 patches (HDP) for stigma pollen loads.

The coefficients of variation for total pollen load and for the proportion of conspecific pollen per pollinator were high (42–82%, calculated on patch pollen load means, Table 3), especially the coefficient of variation (CV) for total pollen load carried by pollinators in high‐density patches (82%).

### Stigma pollen load and the impact of patch structure

At the end of the experiment, we obtained pollen loads from 197 emasculated flowers and 195 control flowers (*N = *100 and 99 individuals, respectively). Conspecific pollen was found on more than 90% of emasculated and 97% of control flowers, and heterospecific pollen was found on 82% of the flowers of each treatment. On average, we found 249.4 ± 25 (SEM) conspecific and 8.8 ± 1 heterospecific pollen grains on emasculated flower stigmas (overall mean 258.2 ± 25, Table 2). On intact flower stigmas, we found 540.7 ± 44.5 conspecific and 5.6 ± 0.7 heterospecific pollen grains (overall mean 546.3 ± 44.8, Table 2). Therefore, on average, flower‐visiting insects provide only about half the total pollen load of the stigma. The proportion of conspecific pollen was high on each stigma, reaching a mean value of 83.3 ± 2.2% for emasculated flowers and 94.8 ± 1.2% for intact flowers (Table 2).

Total pollen quantity in the stigma pollen loads from emasculated and control flowers was not affected by patch density, patch area, or patch visitation rate (Table 4). The proportion of conspecific pollen deposited on emasculated flowers decreased significantly with decreasing patch density and patch area, whereas no such effect was observed in control flowers (Table 4). Visitation rate and interactions between factors had no effects on pollen load size or the proportion of conspecific pollen.

**Table 4:** The effect of patch density, patch area, visitation rate, and their interaction (fixed effects) on stigma pollen load response variables: total number of pollen grains and the proportion of conspecific pollen per stigma

	Total pollen load		Proportion of conspecific pollen	
	*F*_1,21_	*P*‐value	*F*_1,24_	*P*‐value
Emasculated flowers				
Patch density	0.56	0.462	11.38	0.003 (+)
Patch area	1.18	0.290	5.71	0.025 (+)
Visitation rate	3.09	0.093	–	–
Density × area × visitation rate	2.69	0.116	–	–
Intact flowers				
Patch density	0.35	0.56	0.31	0.581
Patch area	3.03	0.096	0.06	0.802
Visitation rate	1.36	0.257	–	–
Density × area × visitation rate	0.36	0.553	–	–

Separate analyses were realized for emasculated and intact flowers. Fixed effects were log‐transformed. See Methods for details of the models used and of model selection based on AIC values. *N = *27 patches. Trends (positive or negative relationship) are shown in parentheses for significant relationships (*P‐*values < 0.05). Degrees of freedom are shown in subscript. Fixed effects not selected based on AIC values are indicated with a dash.

Analyses comparing low‐ and high‐density patches showed that the stigma pollen loads of intact control flowers were not affected by patch density. By contrast, the quantity and quality of emasculated flower stigma pollen loads were 27 and 20% lower, respectively, in low‐density patches (Fig. 1B, D and Table 3). The CV of the total amount of pollen on stigmas was much higher than that of the proportion of conspecific pollen (Table 3) in both emasculated and control flowers. The CV of the total amount of pollen of both emasculated and control flowers was higher in low‐density than in high‐density patches. We found also higher CV for the proportion of conspecific pollen on stigmas from emasculated flowers only, in low‐density than in high‐density patches. The quality of the pollen load of control flowers displayed the lowest levels of variability in both low‐ and high‐density patches (Table 3).

### Relationships between stigma and insect pollen loads

Comparing pollen loads from the same patch, the insect pollen load was much larger than the stigma pollen load (8 times higher, on average, for intact flowers and 17 times higher, on average, for emasculated flowers; Table 2). The total amount of pollen on the stigma of emasculated flowers (*F*_1,16_ = 0.75; *P *=* *0.40) and control flowers (*F*_1,16_ = 3.01; *P *=* *0.102) was not significantly related to the total amount of pollen in the pollinator pollen load (all bumblebees considered together). The proportion of conspecific pollen carried per insects was not significantly related to the proportion of conspecific pollen found on the stigmas of control flowers (*F*_1,16_ = 0.06; *P *=* *0.82) and marginally related to the proportion of conspecific pollen found on emasculated flowers (*F*_1,16_ = 4.20; *P *=* *0.057).

## Discussion

### Low levels of pollen deposition by pollinators

We compared the pollen loads of the stigmas of emasculated and intact flowers, to quantify actual pollen delivery by foraging insects (Kalisz and Vogler 2003). Our results indicate that the levels of pollen deposition by pollinators were low and variable. The overall stigma pollen load of intact flowers able to self‐pollinate consisted largely of conspecific pollen grains and was twice that of emasculated flowers. Indeed, pollinators contributed only about half the conspecific pollen received by the stigma in this facultative autogamous species. Low levels of conspecific pollen deposition by pollinators usually result in pollen limitation of plant reproduction (Wilcock and Neiland 2002). We previously showed that pollen transfer by pollinators is limiting for seed production in *R. ferrugineum*, in both low‐density and high‐density patches (Delmas et al. 2014b). Interestingly, the overall contribution of the pollinators to stigma pollen loads found here is consistent with the mean outcrossing rate of 50% found using a microsatellite analysis during the 2009 growing season (Delmas et al. 2015). This finding suggests that most of the conspecific pollen deposited by pollinators is outcrossed pollen and that geitonogamy may play a smaller role in the pollination system of *R. ferrugineum* than we previously supposed (Delmas et al. 2014a, 2015).

In general, pollen transfer to stigmas depends on the frequency of visits, pollinator constancy, the placement of the pollen on the body of the pollinator, and the pollinator's grooming activity (Harder 1990; Armbruster et al. 1994; Chittka et al. 1999; Pauw 2006). The combined analysis of stigma and pollinator pollen loads showed that the quality and the quantity of the pollen transported by the bumblebees were only marginally related to the quality and the quantity of pollen deposited on stigmas. For example, pollen loads from emasculated flowers (pollinator‐mediated pollination) had higher proportions of conspecific pollen than overall pollinator pollen loads (83% on stigmas vs. 70% on insects). This discrepancy was particularly marked in low‐density patches (74% on stigmas vs. 50% on insects). This discrepancy between pollinator and stigma pollen loads emphasizes the key roles of pollinator behavior and pollen placement on the body of the pollinator in the deposition of conspecific pollen as discussed below. This finding highlights the importance of studying the pollen loads of pollinators, emasculated and intact flowers, to gain an understanding of the processes behind pollination and mating system patterns.

### Lower quantity and quality of pollen transfer in small sparse patches

In general, plant mate and pollinator availabilities would be expected to covary, because pollinators are known to be attracted to large dense populations (Sih and Baltus 1987; Kunin 1997; Cheptou and Avendano 2006). This pattern would be expected to result in large pollinator and stigma pollen loads with a high proportion of conspecific pollen in large, highly attractive, dense plant populations. In small and sparse plant populations, much lower pollen loads are expected (Waites and Agren 2004). Here, at low patch density, the quality of pollen loads transported by pollinators was lower as well as both the quantity and the quality of pollen loads deposited on emasculated flower stigmas. The higher visitation rates in low‐density patches did not attenuate the potentially deleterious consequences of low mate availability for pollen transfer, as found in other species (Duncan et al. 2004b; Campbell and Husband 2007; Wagenius and Lyon 2010). Similar correlations between pollen loads and plant density have been reported for *Limnanthes douglasii rosea* (Briscoe Runquist 2013) and for experimental plots of *Piriqueta caroliniana,* in which the floral constancy of the pollinators may account for the lack of effect of other co‐flowering species on pollen loads and reproductive success at low plant densities (Feldman 2008).

Pollinator behavior effects on pollen loads may play a key role in the pollen transfer patterns observed here. Pollinators seemed to switch between different co‐flowering species more frequently in low‐density patches, and pollinator constancy may therefore have been higher in the high‐density patches, as widely reported for other species (Waser 1978; Chittka et al. 1999). We also observed greater variation of the proportion of conspecific pollen both transported (pollinators) and deposited (emasculated flowers) in low‐density patches with high visitation rates. Accordingly, we observed that *B. wurfleini pyrenaicus*, transporting a small proportion of conspecific pollen (25.3% on average; Table 2), was collected more frequently in the low‐density patches while *B. soroeensis lectitatus*, transporting a higher proportion of conspecific pollen (92.8% on average; Table 2), was collected more frequently in high‐density patches.

### Self‐pollination buffered the effect of patch fragmentation on stigma pollen loads

Our results demonstrated that the quality and the quantity of pollen deposited by pollinators were affected by patch density, but the stigma pollen loads of intact flowers were very similar across the entire gradient of patch size and density. Self‐pollen deposition increased the quantity of conspecific pollen on stigmas (comparing emasculated and intact pollen loads), up to levels matching the number of available ovules (500 ovules per flower on average as presented in Delmas et al. 2015). Here, self‐pollination therefore largely compensated for the deleterious effects on pollen transfer of plant isolation in small sparse patches and of lower visitation rates in large dense patches. The major contribution of self‐pollen to stigma pollen loads in small sparse patches is entirely consistent with previous results showing that reproductive assurance and selfing rates increase, whereas pollen limitation decreases with increasing patch fragmentation (Delmas et al. 2014a, 2015).

## Conclusion

Overall, our results indicate that pollinators carry abundant pollen, but that they deliver insufficient amounts of the conspecific pollen required for ovule fertilization. Pollen loads were only marginally correlated between pollinators and stigmas. These findings highlight the importance of studying both the transport and receipt of pollen to understand pollen transfer patterns. Patch structure was identified as a major factor explaining pollen transfer in this mass‐flowering species. At high plant density and low visitation rate, pollinators deposited relatively small amounts of pollen with a high proportion of conspecific pollen (pollinator limitation). By contrast, at low plant density and higher visitation rate, pollinators deposited even smaller amounts of pollen and the proportion of conspecific pollen was low (mate limitation). Self‐pollen deposition completely buffered the detrimental effects on pollen transfer of patch fragmentation and pollinator failure.

## Conflict of Interest

None declared.

## Supporting information


**Table S1.** List of *Bombus* species (Hymenoptera) collected on *Rhododendron ferrugineum* L. (Ericaceae) for external pollen load analyses.
