# Supplementary material for: Pollen transfer in fragmented plant populations: insight from the pollen loads of pollinators and stigmas in a mass‐flowering species
Source: Ecol Evol. 2016 Jul 21;6(16):5663–73. doi: 10.1002/ece3.2280 (PMC4983582; doi:10.1002/ece3.2280)
Supplement: Supplementary file 1 — Table S1. List of Bombus species (Hymenoptera) collected on Rhododendron ferrugineum L. (Ericaceae) for external pollen load analyses. [file ECE3-6-5663-s001.docx]

**Supporting information**

**Delmas et al.** Pollen transfer in fragmented plant populations: insight from the pollen loads of pollinators and stigmas in a mass-flowering species

*Ecology and Evolution*

**Table S1.** List of *Bombus* species (Hymenoptera) collected on *Rhododendron ferrugineum* L. (Ericaceae) for external pollen load analyses.

| *Bombus (Bombus) lucorum* (L.)  *Bombus (Kallobombus) soroeensis* (Fabricius) *ssp. lectitatus* (Kruseman) |
| --- |
| *Bombus (Psithyrus) rupestris* (Fabricius)*ssp. vasco* (Lepeletier)  *Bombus (Pyrobombus) monticola* (Smith) *ssp. rondoui* (Vogt) |
| *Bombus (Rhodobombus) mesomelas* (Gerstaecker) |
| *Bombus (Confusibombus) confusus* (Schenck)  *Bombus (Thoracobombus) ruderarius*(Müller) *ssp. ruderarius* (Lepeletier)  *Bombus (Melanobombus) lapidarius* (L.)  *Bombus (Megabombus) hortorum* (L.)  *Bombus (Alpigenobombus) wurfleini* (Radoszkowski) *ssp. pyrenaicus* (Vogt) |
